# Content and Effectiveness of Web-Based Treatments for Online Behavioral Addictions: Systematic Review

**DOI:** 10.2196/36662

**Published:** 2022-09-09

**Authors:** Jennifer J Park, Daniel L King, Laura Wilkinson-Meyers, Simone N Rodda

**Affiliations:** 1 School of Population Health The University of Auckland Auckland New Zealand; 2 College of Education, Psychology & Social Work Flinders University Adelaide Australia; 3 Department of Psychology and Neuroscience Auckland University of Technology Auckland New Zealand

**Keywords:** systematic review, gambling, gaming, internet intervention, pornography, treatment, social media

## Abstract

**Background:**

Very few people seek in-person treatment for online behavioral addictions including gaming and gambling or problems associated with shopping, pornography use, or social media use. Web-based treatments have the potential to address low rates of help seeking due to their convenience, accessibility, and capacity to address barriers to health care access (eg, shame, stigma, cost, and access to expert care). However, web-based treatments for online behavioral addictions have not been systematically evaluated.

**Objective:**

This review aimed to systematically describe the content of web-based treatments for online behavioral addictions and describe their therapeutic effectiveness on symptom severity and consumption behavior.

**Methods:**

A database search of MEDLINE, Embase, PsycInfo, Web of Science, Cochrane Central Register of Controlled Trials, and Google Scholar was conducted in June 2022. Studies were eligible if the study design was a randomized controlled trial or a pre-post study with at least 1 web-based intervention arm for an online behavioral addiction and if the study included the use of a validated measure of problem severity, frequency, or duration of online behavior. Data on change techniques were collected to analyze intervention content, using the Gambling Intervention System of CharacTerization. Quality assessment was conducted using the Effective Public Health Practice Project Quality Assessment Tool.

**Results:**

The review included 12 studies with 15 intervention arms, comprising 7 randomized controlled trials and 5 pre-post studies. The primary focus of interventions was gaming (n=4), followed by internet use inclusive of screen time and smartphone use (n=3), gambling (n=3), and pornography (n=2). A range of different technologies were used to deliver content, including websites (n=6), email (n=2), computer software (n=2), social media messaging (n=1), smartphone app (n=1), virtual reality (n=1), and videoconferencing (n=1). Interventions contained 15 different change techniques with an average of 4 per study. The techniques most frequently administered (>30% of intervention arms) were cognitive restructuring, relapse prevention, motivational enhancement, goal setting, and social support. Assessment of study quality indicated that 7 studies met the criteria for moderate or strong global ratings, but only 8 out of 12 studies evaluated change immediately following the treatment. Across included studies, two-thirds of participants completed after-treatment evaluation, and one-quarter completed follow-up evaluation. After-intervention evaluation indicated reduced severity (5/9, 56%), frequency (2/3, 67%), and duration (3/7, 43%). Follow-up evaluation indicated that 3 pre-post studies for gaming, gambling, and internet use demonstrated reduced severity, frequency, and duration of consumption. At 3-month evaluation, just 1 pre-post study indicated significant change to mental health symptoms.

**Conclusions:**

Web-based treatments for online behavioral addictions use an array of mechanisms to deliver cognitive and behavioral change techniques. Web-based treatments demonstrate promise for short-term reduction in symptoms, duration, or frequency of online addictive behaviors. However, there is limited evidence on the effectiveness of web-based treatments over the longer term due to the absence of controlled trials.

## Introduction

There is growing recognition that some individuals engage in problematic and potentially addictive behaviors across a wide range of online activities, including gaming, gambling, shopping, social media use, and pornography use [1-3]. The *International Classification of Diseases 11th Revision* (ICD-11) includes 2 behavioral addictions associated with gaming and gambling [4,5]. Gambling disorder was the first recognized behavioral addiction and is characterized by gambling to escape negative mood, tolerance, repeated unsuccessful attempts to change, and gambling despite negative consequences. Gambling disorder encompasses both land-based activities as well as online casino gambling and web-based betting on sports and racing, which have increased for adults and adolescents over recent years [6,7]. Gaming disorder has characteristics that are consistent with gambling disorder, but there is less focus on money, chasing losses, and financial impacts of gambling on other people. The ICD-11 describes gaming disorder as a condition involving impaired control (eg, over the onset, duration, frequency, and context of play), increasing prioritization of gaming over other activities and life interests, and continued involvement despite negative consequences (eg, impairment in social, educational, and occupational functioning). Some online behavioral addictions are not yet identified under any diagnostic classification of the ICD-11 (eg, pornography and social media use), and some excessive behaviors may be encapsulated by existing categories (eg, online shopping within compulsive buying disorder). Although the literature on different classes of behavioral addictions is still developing, it is often argued that there is a need for evidence-based interventions and other countermeasures to prevent and reduce problematic use.

The literature on interventions for online behavioral addictions has generally been focused on in-person treatment which is intensive and typically involves 6 or more weekly sessions [8,9]. A recent review of treatment for gaming disorder reported it was predominantly psychotherapeutic, face-to-face, and targeted to those with more severe problems [10]. At the same time, reviews have tended to focus on in-person treatment studies and excluded web-based options as evidenced by a recent Cochrane review on psychological therapies for gambling [9]. The lack of scholarly attention on web-based interventions may be overlooking an important modality that is accessed by many affected by behavioral addictions. Online behavioral addictions reportedly affects between 1% and 3% of the population [11,12], but help-seeking rates are quite low [13,14]. These findings suggest that either few people want or require help to resolve their problem or that available clinical options are not meeting the needs of the population. Help seeking may be impeded by structural issues such as the homogeneity of available treatments, prohibitive cost and accessibility, or individual barriers like depression, introversion, or a preference for self-management [15-20].

Web-based treatment appears to be a viable alternative to in-person treatment and has demonstrated effectiveness in reducing symptom severity and consumption patterns of addictive behaviors [21]. Web-based treatment has the potential to reach a wider group of help seekers, such as those seeking anonymity, to reduce perceived shame and stigma [20]. Web-based options may also be attractive for their relatively lower cost compared to individual sessions or retreats [10] and for their convenience and flexibility [20,22,23]. Furthermore, these options may be optimally positioned in the online environment (ie, at the site where users are experiencing psychological difficulties) despite concerns around the appropriateness of web-based delivery for online problems [24]. Online delivery may occur via email, websites, social media, apps, online calls, instant messaging, and virtual reality and may involve smartphones, laptops, and computers, among other online devices. Currently, it is unknown how each of these diverse options might be leveraged effectively to deliver mental health services or other public health measures to address the problematic use of online activities and applications.

Reviews on treatment for online addictive behaviors have not yet explicitly focused on the mode of intervention delivery. Past treatment reviews have also tended to be narrow in focus and overlooked the wide variability in the scope of online activities. For example, reviews of online behaviors have examined interventions for problems related to gaming [8,10,25-29], cybersex [30], both internet use and gaming [31-33], internet use and smartphone use [34], and general internet addiction or problematic internet use [2,35-38]. Reviews focused on gambling problems have examined the effectiveness of web-based treatment for prevention [39] and treatment [23,40], but these were not restricted to samples of online gamblers. Only 1 previous review has examined web-based treatments specifically for problematic internet use, reporting on 3 studies and without examining the effectiveness of treatment [36]. This review included the search terms “online intervention,” “eIntervention,” “eTherapy,” and “eHealth,” which meant other forms of web-based treatments such as online psychotherapy, psychoeducation, and self-help were overlooked. Given these limitations and that considerable time (ie, 5 years) had passed since the previous review, it was timely to evaluate the content and effectiveness of web-based treatments for online behavioral addictions.

This systematic review aimed to summarize and critique the available literature on web-based treatments for online behavioral addictions. Specifically, this review aimed to do the following: describe the content of web-based treatments inclusive of any intervention type for online gaming, gambling, shopping, pornography use, social media use, smartphone use, or nonspecified online use; and describe the effectiveness of web-based treatments on severity, duration, or frequency of consumption. Although only gaming and gambling are currently recognized as addictive disorders in the ICD-11, the scope of this review was expanded to include other online activities (social media, pornography, and shopping) that have been proposed to share similarities to these disorders and which have been studied using addiction-based approaches [1]. It is acknowledged that, over time, there may be important changes to the classification of these behaviors as disorders, including their status of inclusion in *The Diagnostic and Statistical Manual of Mental Disorders* and ICD nomenclatural systems.

## Methods

This systematic review was registered and published on PROSPERO (International Prospective Register of Systematic Reviews; registration code CRD42021224595) and followed the PRISMA (Preferred Reporting Items for Systematic Reviews and Meta-Analyses) guidelines [41].

### Eligibility Criteria

Studies were selected on the basis of the following six inclusion criteria: (1) at least 1 intervention arm was web-based; (2) the behavioral addiction was predominantly a web-based activity and involved gaming, gambling, shopping, pornography use, social media use, smartphone use, or nonspecified internet use; (3) the intervention was intended to reduce the severity, frequency, or duration of the behavioral addiction inclusive of mild and moderate problems; (4) the behavioral addiction was assessed with a validated screen, self-report, or participant registration in a treatment program; (5) the study had a comparison group including a passive or active control or comparative intervention, or was a pre-post study; and (6) there was at least 1 evaluation conducted after the intervention. Unpublished reports, conference papers, presentations, theses, posters, opinion pieces, letters, or protocols were excluded. Studies were also excluded based on the following four criteria: (1) interventions not targeted at web-based behaviors, such as land-based electronic gaming-machine gambling; (2) web-based behavior considered to not be addictive (eg, cyberbullying); (3) prevention programs designed to reduce the risk of future harm or where there were no reported problems; and (4) where the majority of the intervention content was not web-based.

### Identification and Selection of Studies

A database search of MEDLINE, Embase, PsycInfo, Web of Science, Cochrane Central Register of Controlled Trials, and Google Scholar was conducted in June 2022. The search strategy is provided in Multimedia Appendix 1. The search was limited to studies in English language, published in the last 22 years (ie, 2000-2022), and available in full text. To identify potential studies that met the inclusion criteria, recent systematic reviews, reference lists within these reviews, and reference lists of included studies were also searched. Titles and abstracts of the studies returned from the search strategy were screened independently by 2 researchers (JJP and another researcher) against the inclusion and exclusion criteria. The full text of the studies returned from this process was also screened independently by the 2 aforementioned researchers with a third researcher (SNR) involved to resolve any disagreements.

### Data Extraction and Analysis

A structured data extraction form was developed for the study in Microsoft Excel. The data extraction included information on the behavioral addiction type; recruitment and study methods; participant demographics; outcome measures; intervention characteristics; mode of intervention delivery; comparison conditions; and outcomes for frequency, duration, severity, and mental health. To systematically identify the content of interventions, each paper was assessed against the 18 categories of change techniques identified in the Gambling Intervention System of CharacTerization (GIST-1) [42]. The GIST-1 provides an efficient way to classify change techniques sourced from published articles as opposed to assessing the smaller behavior change techniques reported in treatment manuals [43]. Two independent coders (JJP and SNR) assessed each article for the presence of the 18 GIST-1 categories and extracted qualitative data describing each technique.

### Quality Assessment

Each study was assessed for quality using the Effective Public Health Practice Project (EPHPP) Quality Assessment Tool for Quantitative Studies [44]. The EPHPP assesses each study for selection bias, study design, confounders, blinding, data collection method, and study attrition. Each component was rated as strong, moderate, or weak by 2 independent reviewers (JJP and SNR). Each included study was then given a global rating of strong (no weak ratings), moderate (1 weak rating), or weak (2 or more weak ratings).

## Results

### Search Results and Flow Diagram

**Figure 1:**
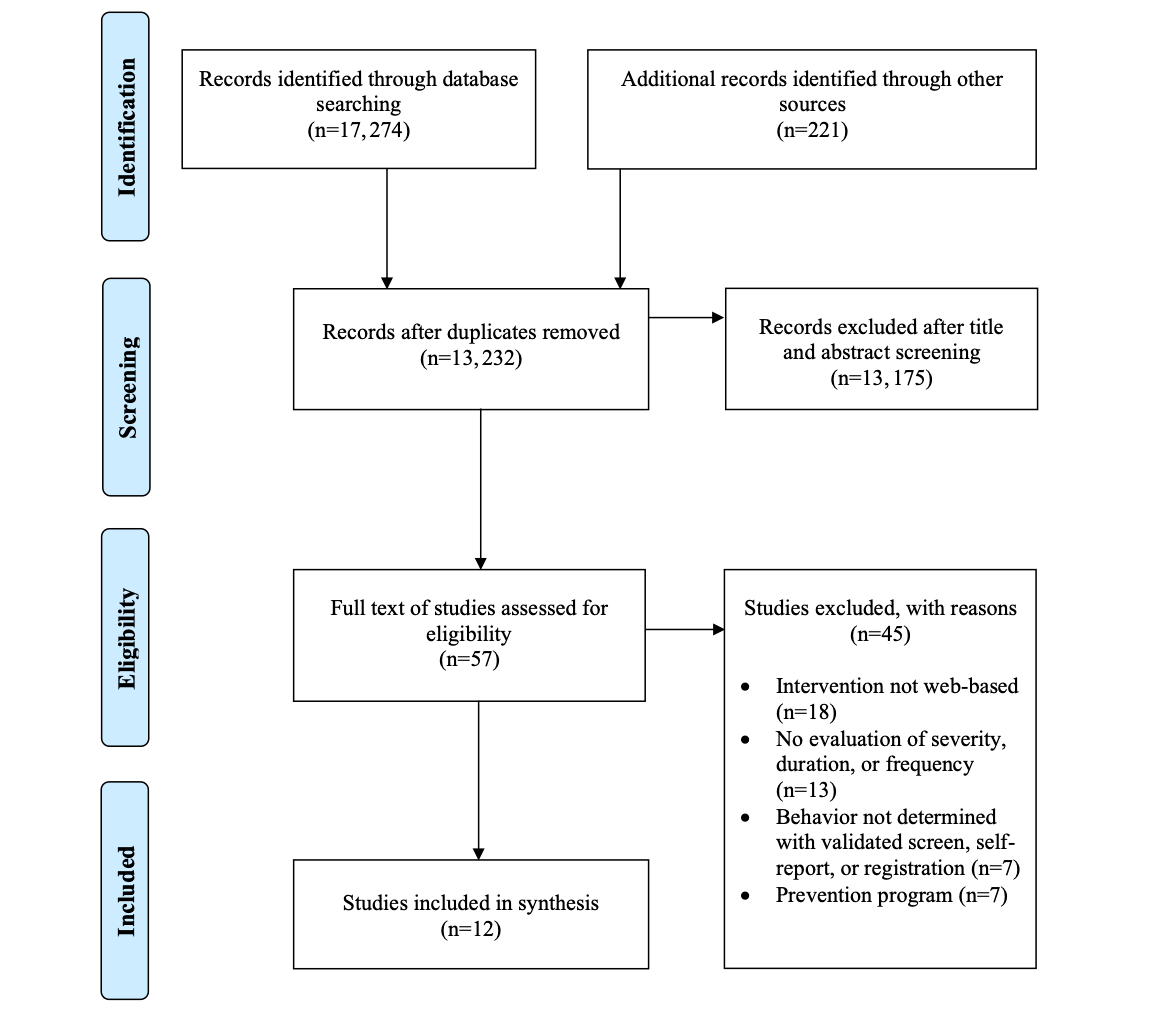
PRISMA (Preferred Reporting Items for Systematic Reviews and Meta-Analyses) flow diagram of study selection.

The search yielded a total of 17,274 studies which included the results of the following 6 databases: MEDLINE (n=2448), Embase (n=3410), PsycInfo (n=2872), Web of Science (n=5750), Cochrane Central Register of Controlled Trials (n=2630), and Google Scholar (n=164). After accounting for duplicates, there were 13,232 studies remaining, of which 13,175 were removed following the review of the title and abstracts of studies against the inclusion criteria (see Figure 1). There was a high number of records requiring screening because of terms such as “internet” and “social media.” The remaining 57 studies were reviewed in full to examine their eligibility for inclusion, which excluded 45 studies. A total of 12 studies with 15 intervention arms, published between 2010 and 2021, were identified for inclusion in the review. The included studies reported on 2218 participants, with individual study sample sizes ranging from 10 to 1122 (mean 184.8, SD 294.3).

### Study Characteristics

Multimedia Appendix 2 presents a summary of included studies. Of the 12 included studies, 7 were randomized controlled trials (RCTs) and 5 were pre-post studies without randomization. Studies recruited participants from Europe (n=8), Asia (n=4), North America (n=3), and Oceania (n=1). Studies predominantly recruited from the community (n=10) via social media, treatment or industry websites, online panels, or online message boards. The primary focus of interventions was gaming (n=4), followed by internet use inclusive of screentime and smartphone use (n=3), gambling (n=3), and pornography (n=2). A range of different technologies were used to deliver content, including websites (n=6), email (n=2), computer software (n=2), social media messaging (n=1), smartphone app (n=1), virtual reality (n=1), and videoconferencing (n=1).

The average age of participants was 33.9 (SD 10.9) years old, and the percentage of males ranged from 10% to 100% (mean 71.8%, SD 31.7%). Almost all participants met the cutoff for problematic behavior, with 8 studies including only people with current problems and 3 studies reporting that the majority had a problem (70%-92%). Participant engagement with the addictive behavior was reported for duration (sessions, days, and weeks) as well as frequency. The average session duration was 57 minutes [45,46], and when measured over 1 week, the average was 27 hours [47-51]. There were 2 studies for internet reduction that reported an average of 5.5 hours of screen time per day [52,53]. The average frequency of engagement was 6 times per week [20,45,46], with 2 studies involving gamblers reporting a frequency of 13 times a month for internet gambling [54] and another study reporting 62 sessions a month for online poker [55].

### Intervention Content

Intervention content was examined in 15 web-based intervention arms from the 12 included studies. To determine the exact content of interventions, the components were assessed and coded into the GIST-1 framework of change techniques [42]. A total of 17 different change techniques were identified (Multimedia Appendix 3). The average number of change techniques per study was 4, with a range of 1 to 10 different techniques. The change techniques most frequently administered (>30% of arms) were cognitive restructuring, relapse prevention, motivational enhancement, goal setting, and social support. No studies included imaginal desensitization or financial management which were previously identified in the GIST-1.

Eight studies (ten arms) reported the delivery of cognitive restructuring [45,51,54,55], cognitive bias modification (CBM) [47,50], exposure therapy [49], or mindfulness [53]. Cognitive restructuring prompted participants to identify, challenge, and replace automatic negative thoughts associated with gaming, gambling, pornography, and nonspecified internet use. Studies also identified triggers and beliefs mediating the relationship between situations and subsequent addictive behavior. Two studies delivered CBM with the aim of altering automatic responses to gaming stimuli. These studies delivered CBM using a device similar to games, where participants used a joystick to push away gaming cues and pull forward neutral or positive associations. Only 1 study delivered exposure therapy that aimed to reduce gaming via virtual reality technology. Exposure therapy involved repeated exposure to high-risk situations, such as scenes from popular games paired with aversion-inducing noise (eg, siren). Just 1 study included mindfulness activities, which were delivered via messaging across 7 days. Participants were prompted to focus their attention on the present moment through engagement with pleasurable activities, including physical activity, breathing, eating, and letting go of disruptive thoughts.

Six studies (eight arms) delivered problem solving [45,48,52], relapse prevention [45,48,51,55], or social skills training [54]. Problem solving prompted participants to identify high-risk situations or triggers that were barriers to sustained behavior change. Participants were also prompted to develop action plans and if-then plans for addressing barriers to change. Relapse prevention prompted participants to review previous goals and plans on what worked well or needed improvement with the view to make plans and prevent future relapse. Just 1 study delivered social skills training for pornography reduction, which focused on improving communication skills and strengthening relationships.

Five studies (five arms) reported the delivery of stimulus control [46,53,56], behavioral substitution [45], or self-monitoring [45,48], and five studies (five arms) delivered social support [45,48,52,54,55]. Stimulus control involved periods of exclusion from online gambling venues or reducing prompts, inclusive of removing notifications, placing the phone out of sight, or turning it off and establishing phone-free periods during the day (eg, before sleep). Conversely, behavioral substitution involved adding pleasurable activities into everyday life. Self-monitoring involved tracking consumption or mood against a self-constructed plan. Social support was provided by clinical or nonclinical professionals who prompted engagement with the intervention and, in 2 cases, delivered the content via videoconferencing or email. Just 2 studies provided peer support, with 1 offering online forums and another integrating fictional characters discussing lived experience within cognitive behavioral therapy (CBT) modules.

Five studies (six arms) reported the delivery of motivational enhancement [45,48,51,52], decisional balance [45,48,51], or goal setting [45,48,51,53]. Motivational enhancement aimed to reduce consumption or increase help seeking by increasing readiness to change. Studies administered motivational interviewing techniques through person-to-person exchanges via videoconferences or nonclinical project support. Motivational enhancement was included as the first module of CBT in 1 study, and another study assessed readiness as a method of tailoring CBT. Three of the studies administering motivational enhancement also offered decisional balance where participants considered the advantages and disadvantages of consumption and reasons for change. Studies that included a goal setting activity prompted participants to establish goal intentions, inclusive of frequency and duration of gaming, pornography use, internet use, and smartphone use.

Seven studies (eight arms) reported the delivery of information gathering [51,52,54], information provision [48,52], feedback on assessment [45,51,53,55], or social comparison [51,55]. Information gathering explored the development of the problem, family history, motives for the addictive behavior, past change attempts, and an assessment of comorbid psychiatric disorders. Information provision included guidelines for reduction and tailored information on support options. Feedback on assessment included a single written and visual report on consumption patterns and severity of addiction, and repeated feedback delivered across 7 days. Two studies provided extended assessment feedback to detail how each individual’s results compared with people of similar age and gender.

### Intervention Effectiveness

Intervention effectiveness was determined by change in problem severity, duration of use, or frequency (see Multimedia Appendix 2). As presented in the following sections, the review also examined change in mental health or psychosocial functioning.

#### Problem Severity

Ten studies examined problem severity, including six RCTs and four pre-post studies. Nine studies conducted after-treatment evaluation, where two RCTs and three pre-post studies indicated reduced problem severity for internet use [51,52], gaming [48], pornography use [45], and smartphone use [53]. One study compared web-based exposure therapy against in-person CBT and reported a reduction in symptom severity and no difference between treatments [49]. Three studies reported no change in internet gambling [46,55] or gaming [47] after treatment. Five studies conducted follow-up evaluation, where three pre-post studies reported reduced severity of gaming [48], gambling [56], and internet use [52]. Two studies reported no change in severity of internet gambling at follow-up evaluation [46,55].

#### Duration

Eight studies assessed duration, including four RCTs and four pre-post studies. Seven studies conducted after-treatment evaluation, where two RCTs reported reduced duration compared with a control group for gaming [50] and internet use [51], and one pre-post study indicated reduced internet use [52]; the remaining four studies indicated no change in duration after treatment [45,46,53] or did not measure change immediately after treatment [48]. Four studies conducted follow-up evaluation, where three pre-post studies reported reduced duration of internet use [52], gaming [48], and gambling [56]. One internet gambling reduction study reported no change in duration at follow-up evaluation [46].

#### Frequency

Five studies assessed frequency, including two RCTs and three pre-post studies. Three studies conducted after-treatment evaluation, where one RCT and one pre-post study indicated reduced frequency of pornography use [45,54]. One study reported no change [55] or did not measure frequency immediately after treatment [48,56]. Three studies conducted follow-up evaluation, where two pre-post studies indicated reduced frequency of gaming [48] and gambling [56]. One RCT indicated no change in frequency of internet gambling at follow-up evaluation [55].

#### Mental Health

Three studies assessed mental health or psychosocial functioning, including one RCT and two pre-post studies. One RCT for gaming demonstrated a reduction in anxiety symptoms after treatment, but not for depression [47]. Two pre-post studies demonstrated an increase in well-being for smartphone use and gaming [48,53] and a reduction in psychological distress for gaming [48]. One pre-post study for gaming conducted follow-up evaluation which indicated improved psychological distress and well-being [48].

### Assessment of Study Quality

On the EPHPP Quality Assessment Tool, 7 out of 12 studies scored a “moderate” or “strong” global rating (see Multimedia Appendix 2). There were 4 studies that had a “weak” global rating due to selection bias, confounds, and low study retention. Just 2 of 12 studies had an associated protocol or registered their study with a trials board [45,55]. Participant retention after treatment was 64.8% (SD 37.5%) with a range of 11% to 100%. The lowest retention was found in 2 gambling studies with 1 on web-based self-exclusion (11%) and 1 delivering CBT to online poker players who were not actively seeking help (15%). One study administering motivational interviewing by videoconferencing reported that almost half of study participants did not complete after treatment evaluation. Only 5 of 12 studies completed follow-up evaluation that was most frequently 3 months [46,48,52,55], with 1 study conducting a 12-month follow-up evaluation [56]. The average follow-up retention rate was 24.0% (SD 30.7%) with a range of 8% to 70%.

## Discussion

This systematic review aimed to summarize and critique the available literature on web-based treatments for online behavioral addictions. The review described and evaluated 12 studies that administered web-based treatments for problems related to online gaming, gambling, pornography, and internet or smartphone use. Treatment was delivered via a range of different technologies inclusive of websites, email, computer software, social media messaging, smartphone apps, virtual reality, and videoconferencing. Treatment delivered an average of 4 different change techniques and, like previous studies involving in-person treatment [10,26,35,42], the most-employed change techniques were cognitive restructuring, relapse prevention, motivational enhancement, goal setting, and social support. The least-used techniques have demonstrated effectiveness for other addictive behaviors or in-person delivery, including exposure therapy, social comparison, feedback on assessment, self-monitoring, and mindfulness. These findings suggest an opportunity to enhance or develop new intervention types that incorporate these techniques.

This review described the effectiveness of web-based treatments on severity, duration, or frequency of consumption. Immediately following treatment, participant evaluation indicated that 5 out of 9 studies that evaluated problem severity reported significant improvements for gaming, pornography, and internet or smartphone use. Out of 7 studies that conducted after-treatment evaluation of duration, 3 reported reduced gaming and internet use. Out of 3 studies that conducted after-treatment evaluation of frequency, 2 reported reduced pornography use. Just 5 of 12 studies conducted follow-up evaluation, and this was most frequently 3 months with one 12-month evaluation. Follow-up indicated that treatment was effective at improving symptoms, duration, or frequency. However, 4 out of 5 studies that conducted follow-up evaluation included completers only, with just 1 RCT for gambling [55] using intent-to-treat analysis which indicated no effect of the intervention. Taken together, this evidence suggests findings should be treated with caution given studies retained just 1 in 4 participants. Easy access is related to high attrition rates because people can easily step away from treatment without interacting with another person [57,58]. One pre-post study on gaming [48] reported a retention rate of 70%, and this study had addressed the risk of attrition by including a coach for advice and support during engagement with the intervention. Future studies should investigate methods such as support or other mechanisms like incentives for increasing retention in web-based treatment for online addictions.

Participants in the included studies were predominantly male and aged around 25 years old. Research indicates that being male and more frequently engaged in addictive online activities is associated with an increased risk of online addictions (gaming disorder, gambling disorder, compulsive buying disorder, and issues related to pornography use and social media) [59], which suggests that most online interventions had appropriate target groups. Participants reported various online intervention components (not content) that were important or helpful, such as privacy and convenience when accessing the intervention, staying engaged with the intervention instead of being overwhelmed or bored, and staying connected to professional support systems through online messaging [48,60,61]. This aligns with research reporting that help seekers have preferences for web-based treatments due to their convenience, accessibility, time efficiency, and ability to connect with professional support in a nontraditional manner (ie, not face-to-face) [20].

The majority of included studies recruited participants from Europe, North America, and Oceania. Only 4 studies recruited from Asian countries despite a significant amount of in-person intervention research for online addictions being conducted in East Asia [62,63]. It bears noting, however, that East Asian studies may often be published in non–English language journals. In South Korea and China, there have been parallel developments in structural and technological restrictions, including content filters, shutdown features, and time limits [64-67]. However, research shows that people experiencing problems with their online addictive behaviors (specifically gaming, in this case) report disapproval with modifications to the structure of activities. Instead, they report stronger support for education, free online screening, self-monitoring tools, and warning labels [11] that are online in nature or can be adapted to be delivered online.

Several limitations of the review should be considered. First, a meta-analysis could not be conducted due to the limited quality of studies, limited data available, and varying study designs. Second, just 7 RCTs were included, but only 3 conducted short- or medium-term follow-up evaluation. In addition, the findings from the included studies were limited due to high rates of attrition at follow-up evaluation, with 4 out of 5 studies reporting on completer analysis only. To determine the effectiveness of web-based treatment, there needs to be a greater focus on RCT study design as well as participant retention and long-term follow-up evaluation. Third, we did not include non–English language literature, which might have excluded a large body of research conducted in East Asia. A strength of our study was the inclusion of studies that were focused on treatment rather than on prevention or early intervention; however, due to the heterogenous study focus and design, we were unable to determine who would likely benefit from web-based treatment. Future studies might consider examining the effectiveness of web-based treatment, level of problem severity, and type of addictive behavior. Just 1 included study compared in-person and web-based treatment and reported significant improvements in symptom severity after 8 sessions with no difference between groups. If future research finds web-based outcomes are similar to in-person treatment, then there is a strong case for expansion of web-based options.

There were also several limitations in relation to describing the content of interventions. We used the GIST-1 [42] to categorize change techniques instead of the 93-item behavior change technique (BCT) taxonomy [43] because of the absence of associated protocols or study registration. Just 2 studies referenced a published or manualized protocol or trial registration, which may reflect the exploratory nature of the research at this time. The lack of detailed reporting is common in behavioral addictions and was a reason for the development of the GIST-1 classification system which enables researchers to reliably code brief treatment descriptions [42]. Future studies may consider obtaining treatment manuals or working with a study developer to map the content of effective treatments onto the 93-item BCT taxonomy [43]. The current study was also limited to describing the content of interventions because of the limited sample. Future studies should consider examining the relationship between change techniques and participant outcomes. Studies should also consider examining the theoretical underpinnings or mechanisms of interventions and whether these also have an impact on severity, duration, or frequency of use.

This systematic review identified 12 studies assessing web-based treatments for online behavioral addictions. These findings highlight the potential of emerging web-based treatments, but the current evidence base varied greatly in study quality. This review also highlights the importance of having treatment protocols registered or published alongside an article and reporting components as aligned with BCTs or change techniques to be able to replicate studies with the exact components. Enhanced research designs are needed to develop a stronger evidence base to inform health care guidelines. Future research should also consider the relative appropriateness and cost-effectiveness of web-based treatments to guide the provision and allocation of funding across health systems. The review should be updated as more evidence on intervention effectiveness across online behavior types becomes available.

## Appendix

Multimedia Appendix 1 Search strategy.

Multimedia Appendix 2 Characteristics of included studies (N=12).

Multimedia Appendix 3 Content of web-based treatment in included studies (N=12).

## Abbreviations

BCT: behavior change technique
CBM: cognitive bias modification
CBT: cognitive behavioral therapy
EPHPP: Effective Public Health Practice Project
GIST-1: Gambling Intervention System of CharacTerization
ICD-11: International Classification of Diseases 11th Revision
PRISMA: Preferred Reporting Items for Systematic Reviews and Meta-Analyses
PROSPERO: International Prospective Register of Systematic Reviews
RCT: randomized controlled trial
