# Supplementary material for: Content and Effectiveness of Web-Based Treatments for Online Behavioral Addictions: Systematic Review
Source: JMIR Ment Health. 2022 Sep 9;9(9):e36662. doi: 10.2196/36662 (PMC9508667; doi:10.2196/36662)
Supplement: Multimedia Appendix 1 [file mental_v9i9e36662_app1.docx]

**Multimedia Appendix 1: Search strategy**

(Excessive OR problem* OR compulsi* OR addict* OR patholog*)

AND (“internet” OR “internet-based” OR “online” OR “on-line” OR “electronic” OR “technolog*” OR “web” OR “web-based” OR “website?” OR “video*” OR “digital*” OR “phone?” OR “smartphone?” OR “mobile” OR “text message*” OR “texting” OR “messaging” OR “computer?” OR “laptop?” OR “tablet?” OR “cyberspace” OR “cyber space”)

AND (“gaming” OR “video game” OR “videogame*” OR “screen time” OR “computer game” “gambl*” OR “betting” OR “sport*” OR “bingo” OR “social media” OR “social network*” OR “shopping” OR “buying” OR “porn*” OR “cybersex” OR “sext*” OR “erotic*” OR “hypersex*”)

AND (“intervention*” OR “treatment*” OR “brief intervention” OR “psychotherap*” OR “psychoeducation*” OR “therap*” OR “recovery” OR “self exclu*” OR “self-exclu*” OR “feedback” OR “harm minimisation” OR “harm minimization” OR “self-help” OR “self help” “self-manage*” OR “self manage*” OR “self-evaluat*” OR “self evaluat*” OR “self-monitor” OR “self monitor” OR “self-regulat*” OR “self regulat*” OR “telemedicine” OR “telepsychiatry” OR “telepsychology” OR “mobile health” OR “mhealth” OR “m-health”)

Note: MeSH terms for MEDLINE and PsycInfo, and Emtree terms for Embase were used in combination with the search strategy.
