# Supplementary material for: Content and Effectiveness of Web-Based Treatments for Online Behavioral Addictions: Systematic Review
Source: JMIR Ment Health. 2022 Sep 9;9(9):e36662. doi: 10.2196/36662 (PMC9508667; doi:10.2196/36662)
Supplement: Multimedia Appendix 2 [file mental_v9i9e36662_app2.docx]

**Multimedia Appendix 2: Characteristics of included studies (n=12)**

| **Study ID** | **Country and setting** | **Target and design** | **Sample size, age, and sex** | **Problem-related inclusion criteria** | **Outcome measures^a^** | **Direction of group effects^b^** | **EPHPP^c^**  **and retention** |
| --- | --- | --- | --- | --- | --- | --- | --- |
| Bőthe et al [45] | USA, UK, Canada, Hungary, India  Community advertising and social media | Pornography  RCT | N=264; Mean=33.2 years (SD=10.6); 96.2% male. | Problematic pornography use | Post at 6 weeks  Frequency: Pornography use in the past 7 days.  Duration: Time per session.  Severity: Problematic Pornography Consumption Scale (PPCS). | Frequency: +ve.  Duration: No effect.  Severity: +ve. | Moderate  35% retention |
| Bottel et al [52] | Germany  Community advertising and social media | Internet  Pre-post | N=140; Mean=33.2 years (SD=13.3); 85% male. | Internet Use Disorder based on DSM-5 for Internet Gaming Disorder (IGD) | Post after 2 sessions and follow-up at 3 months  Duration: Time spent using the internet.  Severity: Short Internet Addiction Test (s-IAT). | Duration: +ve at all time points.  Severity: +ve at all time points. | Weak  52% retention at post and 9% at follow-up. |
| Caillon et al [46] | France  Community advertising and online panel | Internet gambling  RCT | N=60; Mean= 35.2 years^d^;  73% male | Moderate risk on Problem Gambling Severity Index (PGSI) and gambling at least once during the past month | Post at day 15 and follow-up at 2 months  Duration: Time spent gambling in the past 7 days.  Severity: PGSI. | Duration: No effect at any time points.  Severity: No effect at 2 months. | Weak  Retention NR |
| Hardy et al [54] | USA  Current treatment seekers online | Pornography  Pre-post | N=138; Mean=37.97 years (SD=12.4);  97% male | Current treatment-seekers enrolled in the intervention | Post only  Frequency: Pornography use a month prior to and since starting treatment. | Frequency: +ve. | Weak  25% retention |
| Hayer et al [56] | Austria  Community recruitment from gambling website | Internet gambling  Pre-post | N=259; Mean=36.2 years^d^;  68% male | Gambling website users that choose to self-exclude | Follow-up at 1 month, 6 months and 12 months after exclusion  Frequency: Number of gambling days.  Duration: Time spent gambling.  Severity: Lie-Bet Questionnaire. | Frequency: +ve at all time points.  Duration: +ve at all time points.  Severity: +ve at all time points. | Weak  11% retention at 1 month, 9% at 6 months, and 8% at 12 months |
| He et al [47] | China  Community recruitment from university | Gaming  RCT | N=48; Mean=20.0 years (SD=1.95); 81% female | IGD severity scale score ≥ 32 (based on DSM-5) and gaming time >2 hours per day for more than a year | Post at day 5  Severity: Internet Gaming Disorder based on DSM-5. | Severity: No effect. | Moderate  100% retention |
| Kent et al [53] | UK  Community recruitment from university | Smartphone  Pre-post | N=10; Mean= 21.7 years (SD=3.47); 90% female | Problematic online use score > 30 on the Internet Addiction Test (IAT) | Post at day 62  Duration: Screen time per day.  Severity: Mobile Phone Problem Use Scale (MPPUS) and IAT. | Duration: No effect.  Severity: +ve. | Moderate  100% retention |
| Luquiens et al [55] | France  Community recruitment from gambling website | Internet gambling  RCT | N=1122; Mean=34.7 years (SD=10.1);  92% male | PGSI score ≥5 | Post at 6 weeks and follow-up at 12 weeks  Frequency: Number of gambling sessions in the past 3 days.  Severity: PGSI. | Frequency: No effect for any intervention arms.  Severity: No effect for any intervention arms. | Weak  15% retention at post and 8% at follow-up |
| Park et al [48] | New Zealand  Community advertising via flyers and social media | Gaming  Pre-post | N=50, Mean=25.0 years (SD=4.79); 90% male | People who want to reduce their gaming in the next 30 days | Post at day 30 and follow-up at 3 months  Frequency: Number of gaming sessions in a week.  Duration: Hours spent gaming in a week.  Severity: Game Addiction Scale (GAS) and Orzack Time-Intensity Scale. | Frequency: +ve at 3 months.  Duration: +ve at 3 months.  Severity: +ve at 3 months for GAS, +ve at all time points for Orzack Time-Intensity Scale. | Moderate  84% retention at post and 70% at follow-up |
| Park et al [49] | South Korea  One university medical center | Gaming  RCT | N=24; Mean=23.7 years (SD=2.9);  100% male | Gaming time >30 hours per week, Young’s Internet Addiction Scale (YIAS) score >50, and the experience of negative consequences. | Post after 8 sessions  Severity: YIAS. | Severity: +ve within subjects change and no effect between groups. | Moderate  100% retention |
| Rabinovitz et al [50] | Israel  Community recruitment from online gaming groups and forums | Gaming  RCT | N=38; Mean=22.8 years (SD=3.96);  100% male | Massively multiplayer online role-playing game time >10 hours a week for at least 1 year | Post immediately following the intervention  Duration: Intended gaming time. | Duration: +ve. | Moderate  100% retention |
| Su et al [51] | China  Community recruitment from university | Internet  RCT | N=65; NR;  69% female | Young’s Diagnostic Questionnaire (YDQ) score ≥ 5 or score of 3-4, and online time >14 hours per week | Post at 1 month  Duration: Time spent using the internet each day of the week.  Severity: YDQ | Duration: +ve for all intervention arms.  Severity: +ve for all intervention arms. | Strong  91% retention |

^a^ Outcomes shown in table include frequency, duration, and severity. Mental health outcomes are not shown; ^b^ Direction of group effects: No effect=p>0.05; +ve=beneficial effect of intervention on outcome; -ve=worsening of outcome in intervention group; ^c^ Effective Public Health Practice Project (quality assessment); ^d^ Standard deviation not reported.
