# Supplementary material for: Content and Effectiveness of Web-Based Treatments for Online Behavioral Addictions: Systematic Review
Source: JMIR Ment Health. 2022 Sep 9;9(9):e36662. doi: 10.2196/36662 (PMC9508667; doi:10.2196/36662)
Supplement: Multimedia Appendix 3 [file mental_v9i9e36662_app3.docx]

**Multimedia Appendix 3: Content of web-based treatment in included studies (n=12)**

| **Study ID** | **Target** | **Intervention characteristics** | **1** | **2** | **3** | **4** | **5** | **6** | **7** | **8** | **9** | **10** | **11** | **12** | **13** | **14** | **15** | **16** | **17** |
| --- | --- | --- | --- | --- | --- | --- | --- | --- | --- | --- | --- | --- | --- | --- | --- | --- | --- | --- | --- |
| Bőthe et al [45] | Pornography | 6 modules delivered over 6 weeks plus 1 booster session via website. | ✓ | ✓ | ✓ | ✓ | ✓ |  | ✓ | ✓ |  |  | ✓ | ✓ |  | ✓ |  |  |  |
| Bottel et al [52] | Internet | 2 sessions over 2 weeks via video conferencing. | ✓ |  | ✓ |  |  |  |  |  |  |  |  | ✓ |  |  | ✓ | ✓ |  |
| Caillon et al [46] | Gambling | Self-exclusion for 7 days via website. |  |  |  |  |  |  |  |  |  |  |  |  | ✓ |  |  |  |  |
| Hardy et al [54] | Pornography | 10 modules over 4 months delivered via website. |  |  |  |  |  |  |  | ✓ |  | ✓ |  | ✓ |  |  |  | ✓ |  |
| Hayer et al [56] | Gambling | Self-exclusion for 1, 3, 6 or 12 months via website. |  |  |  |  |  |  |  |  |  |  |  |  | ✓ |  |  |  |  |
| He et al [47] | Gaming | 4 sessions over 4 days delivered via software. |  |  |  |  |  |  |  | ✓ |  |  |  |  |  |  |  |  |  |
| Kent et al [53] | Smartphone | 1 goal setting module plus 21 messages over 3 weeks via smartphone app. |  |  |  | ✓ | ✓ |  |  |  |  |  |  |  | ✓ |  |  |  | ✓ |
| Luquiens [55] | Gambling | 6 modules over 6 weeks with guidance. |  |  |  |  |  |  | ✓ | ✓ |  |  |  | ✓ |  |  |  |  |  |
|  |  | 6 modules over 6 weeks without guidance. |  |  |  |  |  |  | ✓ | ✓ |  |  |  |  |  |  |  |  |  |
|  |  | 1 session of personalized normative feedback via email. |  |  |  |  | ✓ | ✓ |  |  |  |  |  |  |  |  |  |  |  |
| Park et al [48] | Gaming | 1 session delivered via website with support offered 3 times over 30 days via email or social media. | ✓ | ✓ | ✓ | ✓ |  |  | ✓ |  |  |  | ✓ | ✓ |  |  | ✓ |  |  |
| Park et al [49] | Gaming | 8 sessions of exposure therapy over 4 weeks via virtual reality technology. |  |  |  |  |  |  |  | ✓ | ✓ |  |  |  |  |  |  |  |  |
| Rabinovitz et al [50] | Gaming | 1 session of cognitive bias modification in 1 day via software. |  |  |  |  |  |  |  | ✓ |  |  |  |  |  |  |  |  |  |
| Su et al [51] | Internet | 4 modules with a single session of normative feedback delivered in 1 day (lab) or over 1 week (home) via website. | ✓ | ✓ |  | ✓ | ✓ | ✓ | ✓ | ✓ |  |  |  |  |  |  | ✓ | ✓ |  |
|  |  | 4 modules without normative feedback delivered in 1 day (lab) via website. | ✓ | ✓ |  | ✓ |  |  | ✓ | ✓ |  |  |  |  |  |  | ✓ | ✓ |  |

1 Motivational enhancement; 2 Decisional balance; 3 Problem solving; 4 Goal setting; 5 Feedback on assessment; 6 Social comparison; 7 Relapse prevention; 8 Cognitive restructuring; 9 Exposure therapy; 10 Social skills training; 11 Self-monitoring; 12 Social support; 13 Stimulus control; 14 Behavioral substitution; 15 Information provision; 16 Information gathering; 17 Mindfulness
